# β-Caryophyllene as a Novel Modulator of the Renin–Angiotensin System: A Path to Reduce Inflammation and Restore Taste Function

**DOI:** 10.3390/biomedicines13102514

**Published:** 2025-10-15

**Authors:** Sofía Cecilia López-Salido, Hugo Alejandro Espinoza-Gutiérrez, Mario Eduardo Flores-Soto, Alma Hortensia Martínez-Preciado, Juan Manuel Viveros-Paredes

**Affiliations:** 1 Laboratorio de Investigación y Desarrollo Farmacéutico, Departamento de Farmacobiología, Centro Universitario de Ciencias Exactas e Ingenierías, Universidad de Guadalajara, Guadalajara 44430, Mexico; sofia.lopez8801@alumnos.udg.mx (S.C.L.-S.); hugo.espinoza2323@alumnos.udg.mx (H.A.E.-G.); 2 Laboratorio de Neurobiología Celular y Molecular, División de Neurociencias, Centro de Investigación Biomédica de Occidente, Instituto Mexicano del Seguro Social, Guadalajara 44340, Mexico; mariosoto924@yahoo.com.mx; 3 Laboratorio de Ingeniería y Biotecnología de los Alimentos, Departamento de Ingeniería Química, Centro Universitario de Ciencias Exactas e Ingenierías, Universidad de Guadalajara, Guadalajara 44430, Mexico; hortensia.martinez@academicos.udg.mx

**Keywords:** dysgeusia, β-Caryophyllene, renin-angiotensin system, inflammation, apoptosis, ACE2, CB2

## Abstract

**Background/Objectives**: Dysgeusia is a taste disorder commonly associated with chronic inflammation, reducing the quality of life, particularly in ageing populations or individuals with non-communicable chronic diseases. This study aimed to evaluate the effect of β-Caryophyllene, a natural sesquiterpene and agonist of the cannabinoid receptor 2 (CB2), on dysgeusia through an analysis of inflammation, Renin–Angiotensin System (RAS) and taste perception. **Methods**: Male BALB/c mice were subjected to a dysgeusia model induced by molecular mimicry with lipopolysaccharide. Animals received intraperitoneal injections of lipopolysaccharide in a chronic–persistent regimen, starting at a dose of 35 μg/100 g body weight for 7 days until reaching a final concentration of 250 μg/100 g and a daily oral administration of β-Caryophyllene at a dose of 10 mg/kg. The effect of β-Caryophyllene on taste perception, inflammatory biomarkers, RAS key-elements, CB2 expression and physiological parameters was evaluated. **Results**: Data indicate that β-Caryophyllene attenuates systemic inflammation by decreasing IL-1β and IL-6 and increasing ACE2 enzymatic activity in lingual tissue. Also, it was shown that the sesquiterpene reduced taste cell apoptosis and improved sucrose preference, suggesting a feasible restoration of taste dysfunction. **Conclusions**: These findings demonstrate that β-Caryophyllene could be a potential candidate for treating dysgeusia due to its putative anti-inflammatory and angiotensinergic effects.

## 1. Introduction

The population of older adults aged 65 and above has been rising recently. This demographic has reached an unprecedented rate of 8.5% of the total population worldwide [1]. This population also exhibits an increased vulnerability to Noncommunicable Chronic Diseases (NCD), a significant global concern accounting for 74.0% of all deaths and 82.0% of premature mortalities worldwide [2]. These two conditions share a common feature: chronic inflammation, a complex process that contributes to the development and progression of various diseases such as type 2 diabetes [3], cardiovascular diseases [4], obesity [5], and cancer [6].

Chronic inflammation manifests a range of symptoms, including the complete loss of taste perception, known as dysgeusia [7]. Dysgeusia significantly impacts life quality for older adults and vulnerable populations with an NCD, affecting their eating habits, nutritional status, and general well-being [8]. The pathophysiology of dysgeusia associated with chronic inflammation is complex and involves various mechanisms. At a local level, in the oral cavity, inflammation affects the function of taste buds, accelerating their degeneration process and reducing their lifespan [9,10]. Inflammation promotes the release of Interleukin-1β (IL-1β), Interleukin-6 (IL-6), and Tumor Necrosis Factor-α (TNF-α). TNF-α not only amplifies the inflammatory cascade but also enhances oxidative stress and downregulates ACE2 expression, thereby exacerbating the imbalance of the Renin–Angiotensin System (RAS) and contributing to the impairment of taste bud function [11]. RAS alteration leads to an increase in Angiotensin II (Ang II), which in turn promotes enhanced vasoconstriction and inflammation through the classical angiotensin-converting enzyme (ACE)/Ang II/angiotensin II type 1 receptor (AT1R) axis [12]. Therefore, the RAS may play a significant role in inflammation, particularly since ACE2 has been found to be expressed in the tongue [12,13].

Addressing the underlying inflammation related to dysgeusia is crucial for potential therapeutic strategies that enhance the health and well-being of this growing demographic. Therapeutic options for dysgeusia are limited and often do not focus on addressing the underlying cause. Conventional treatment usually consists of a balanced diet rich in micronutrients to train taste perception [14,15]. Corticosteroids are occasionally used to reduce systemic inflammation; however, this pharmacological approach is not recommended due to side effects in glucose metabolism [16].

The endocannabinoid system, particularly the CB2 receptor (CB2), is a promising target. Studies have shown that CB2 activation exerts anti-inflammatory and analgesic effects, modulating the immune response and reducing the release of proinflammatory cytokines like IL-1β and IL-6 [17]. On the other hand, the RAS also encompasses counter-regulatory axes, such as the Angiotensin-Converting Enzyme 2 (ACE2)/Angiotensin-(1-7) (Ang-(1-7)) axis, which exerts anti-inflammatory functions [18]. Beyond these, other inflammatory systems also contribute to tissue homeostasis and pathology, such as the NF-kB pathway. At the same time, the NLRP3 inflammasome is a critical regulator of IL-1β and IL-18 release during innate immune responses. Additionally, the oxidative stress pathway, which is mediated through NADPH oxidases, amplifies inflammation [19]. Among these targets, CB2 activation is particularly valuable due to its multiple roles in regulating inflammatory systems.

Phytochemicals with cannabinoidergic and angiotensinergic activity such as β-Caryophyllene [17,20] could be a novel therapeutic strategy to improve taste perception within the framework of dysgeusia. β-Caryophyllene is a natural sesquiterpene predominantly found in black pepper (*Piper nigrum*), hops (*Humulus lupulus*), and *Cannabis sativa* [21]. Moreover, β-Caryophyllene is orally bioavailable and considered safe for use [22]. It has been shown that β-Caryophyllene interacting with CB2R inhibits the MAPK pathway, reducing inflammation and increasing cell proliferation in different cell types [23,24].

The present study aims to evaluate the effect of β-Caryophyllene on the pathophysiology of dysgeusia induced by chronic lipopolysaccharide administration in BALB/c mice by quantifying pro- and anti-inflammatory cytokines, analyzing key components of the counter-regulatory RAS axis such as ACE2 enzymatic activity, and assessing both general health status of the animals and their taste perception.

## 2. Materials and Methods

### 2.1. Animals

A total of fifty (50) male BALB/c mice (8–10 weeks old) were used. Animals were obtained from the Centro de Investigación Biomédica de Occidente (CIBO) of the Instituto Mexicano del Seguro Social (IMSS), Mexico. Mice were housed in groups of five in standard polycarbonate cages 20 × 60 × 40 cm (width, length, height) under controlled environmental conditions (23 ± 2 °C, 12 h light/dark cycle). Food and water were provided ad libitum, except during behavioral testing. Animal care and all experimental procedures, including euthanasia, were conducted in accordance with the National Institutes of Health (NIH) Guide for the Care and Use of Laboratory Animals and the recommendations of the ARRIVE guidelines for animal research.

### 2.2. Experimental Design and Drug Administration

Mice were randomly divided into five experimental groups (*n* = 10). The control group (CT) did not receive any treatment The positive control (CT+) was administered orobuccally with 20 μL of a hypertonic solution of zinc sulfate monohydrate (ZnSO_4_·H_2_O) 0.17 M, the last two days of the experimental design (days 6 and 7). This timing was deliberately selected due to the corrosive nature of zinc sulfate, which has the potential to induce elevated stress levels in the subjects if administered over an extended period.

The lipopolysaccharide-insulted group (LPS) received daily intraperitoneal administrations of O111:B4 *Escherichia coli* lipopolysaccharide (Sigma-Aldrich, L2630, St. Louis, MO, USA) for seven consecutive days following a chronic-persistent regimen [25]. Doses began at 35 μg/100 g body weight and increased linearly to 70, 105, 140, 175, 210, and 250 μg/100 g.

**Figure 1:**
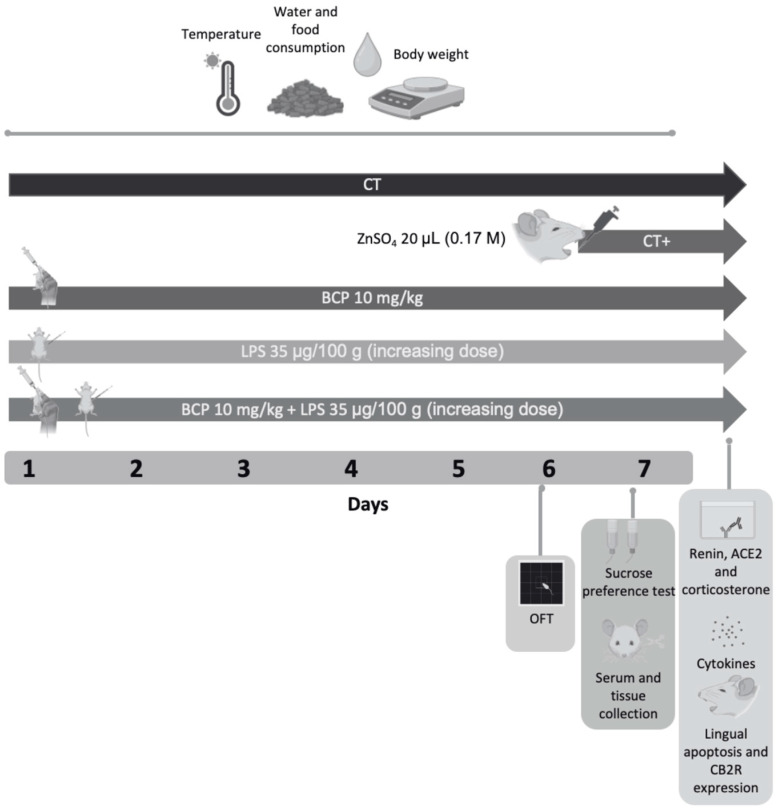
Diagram of the experimental design and drug administration. The study spanned 7 days and included five groups (*n* = 10, each group): Control group (CT), positive control group (CT+), β-Caryophyllene control group (BCP), lipopolysaccharide group (LPS), and therapeutic group (BCP+LPS). On Day 6, the open field test was conducted on all groups. On Day 7, the two-bottle preference test was performed, followed by animal euthanasia and serum/lingual tissue collection. Subsequent analyses included quantification of renin concentration, ACE2 enzymatic activity, pro and anti-inflammatory cytokine levels, corticosterone serum concentration, apoptosis and CB2 expression in lingual tissue. Health status, including food intake, water consumption, body weight and temperature, were monitored throughout the seven days of the experimental design.

The β-Caryophyllene control group (BCP) received an oral dose of 10 mg/kg (body weight) of β-Caryophyllene for seven consecutive days. The in vivo dose of β-Caryophyllene was established based on prior data from our research group [26,27,28], as well as on the foundational study by Gertsch et al. (2008) investigating the cannabinergic properties of β-Caryophyllene [17]. The administration was 30 min before monitoring the health status. The β-Caryophyllene was donated by Prof. Dr. Jürg Gertsch from the Institute of Biochemistry and Molecular Medicine of the University of Bern, Switzerland. The vehicle used is a mix of 4% (*v*/*v*) Tween-80 and sterile saline solution. The therapeutic group (BCP + LPS) along with the oral administration of the β-Caryophyllene, and the intraperitoneal chronic-persistent lipopolysaccharide administration as indicated above. β-Caryophyllene was administered 30 min prior lipopolysaccharide due to pharmacokinetic reasons. A graphical representation of the experimental design is provided in Figure 1.

In accordance with ethical standards for animal experimentation, the study received approval from the Institutional Committee for the Care and Use of Laboratory Animals (CICUAL) of the Centro Universitario de Ciencias Exactas e Ingenierías (CUCEI), Universidad de Guadalajara, under the registration code CUCEI/CINV/CICUAL-06/2023.

### 2.3. Monitoring of Health Status

Health status, including food intake, water consumption, body weight and temperature, were monitored throughout the seven days of the experimental design. Food consumption (g) was determined daily by weighing the food provided to each cage before and after the experimental period. The difference in weight represented the amount of food consumed within 24 h. Similarly, water intake (mL) was assessed by measuring the water provided and subtracting the remaining volume at the end of each day. Body weight (g) was assessed daily. Animals were weighed using a calibrated digital scale. To minimize stress, mice were gently placed in a holding container before weight measurement. Core temperature (°C) was assessed daily throughout the experimental period every 30 min post-administration for 90 min. Baseline measurements were obtained before any experimental manipulations (i.e., administration of ZnSO_4_, lipopolysaccharide, and/or β-Caryophyllene). Temperature assessment was conducted via a temperature-sensitive probe (RET-3 Rectal Probe, Physitemp, Clifton, NJ, USA) connected to a temperature recorder (BAT-12, Physitemp, Clifton, NJ, USA). The probe was inserted rectally to a depth not exceeding 2 cm [29], then the area under the curve (AUC) was calculated, taking into account both positive and negative peaks.

### 2.4. Open Field Test

The primary objective of this test was to assess sickness behavior by analyzing movement patterns. Parameters such as total distance traveled, time spent moving, time spent in the center, and activity levels within the first five min were evaluated, as described by Gould (2011) [30]. Mice were placed in a 40 × 40 × 50 cm arena subdivided into 16 squares (10 × 10 cm, rows A–D, columns 1–4) and allowed a one-minute habituation before being recorded from above for five min. The arena was disinfected with 70% ethanol after each session. Videos were analyzed using ANY-maze version 7.08 (Stoelting Co., Wood Dale, IL, USA), tracking the animals’ center to obtain trajectories, heat maps, total distance (m), mean speed (m/s), and time (s) in corner (A1, A4, D1, D4) or central zones (B2, B3, C2, C3). Behavioral assessments followed a single-blind design: the handler knew the treatments, whereas video analysis was performed by blinded observers.

### 2.5. Two-Bottle Preference Test

A modified two-bottle preference test protocol, adapted from Sinclair et al. [31] was implemented to asses taste perception. Mice were offered a choice between two solutions: a 3% sucrose solution and distilled water. Prior experimental trials, mice underwent a three-day habituation period to acclimate to the testing environment. Habituation sessions were conducted once daily for one hour. On day one, mice were placed in the testing chamber with a single water bottle on the left side. On day two, the water bottle was placed on the right side. Finally, on day three, both bottles (water only) were placed in the chamber. The two-bottle choice paradigm was employed for the experimental trials, with one bottle containing the 3% sucrose solution and the other containing distilled water for 5 min. Fluid consumption (mL) from each bottle was measured and compared to assess taste function.

### 2.6. Collection of Serum and Lingual Samples

Of the ten mice in each experimental group, five were processed fresh for the collection of serum and tongue tissue homogenates. Animals were euthanized via decapitation. No anesthetic was administered; mice were habituated to human handling for 5 days prior t the procedure. Decapitation was performed instantly in a separate area that had been disinfected with 0.5% sodium hypochlorite and 70% ethanol, in order to minimize stress associated with blood odor. Blood samples were collected immediately post-mortem and cryopreserved in test tubes for subsequent analysis. Tongue tissue was also extracted and subjected to a homogenization process by probe sonication at a 1:10 ratio. An antiprotease buffer containing 2-mercaptoethanol was added to the homogenate. The remaining five were perfused intracardially with 10% formaldehyde. Tongues were then dissected, cryopreserved in a 10% formaldehyde solution for one week and then stored in a 40% sucrose solution until further analysis. Total protein concentration of the samples was determined using the Lowry method [32].

### 2.7. Determination of the Enzymatic Activity of ACE2 and Renin Concentration

ACE2 enzymatic activity was assessed using a commercially available fluorometric activity assay kit (Sigma-Aldrich, MAK377, St. Louis, MO, USA). The assay is based on the principle that the intensity of the fluorescent signal is directly proportional to the catalytic activity of the ACE2 enzyme. Fluorescence was quantified at baseline and after 60 min (excitation 320 nm, emission 420 nm) using a Synergy HT microplate reader (BioTek Instruments, Winooski, VT, USA) in duplicate. Data were expressed as picomoles per minute per microgram of protein (pmol/min/μg).

Renin concentration was determined using a commercially available ELISA kit (Sigma-Aldrich, RAB0565-1KT, St. Louis, MO, USA). This assay employs a capture antibody that binds to the target antigen (renin). A secondary antibody is then added, which generates a detectable signal upon enzymatic reaction with a substrate. The assay was carried out at a wavelength of 450 nm with a Multiskan GO microplate spectrophotometer (Thermo Scientific, 51119200, Waltham, MA, USA) in duplicate. Data were expressed as micrograms of renin per milligram of tissue (μg/mg) and micrograms of renin per milliliter of serum (μg/mL). All procedures were performed following the manufacturer’s instructions.

### 2.8. Quantification of Pro and Anti-Inflammatory Cytokines

A magnetic bead-based multiplex immunoassay was employed to quantify multiple cytokines simultaneously (Merck Millipore, MCYTOMAG-70K, Burlington, MA, USA). The assay utilizes antibody-conjugated magnetic beads to capture and quantify target analytes within a single well. The beads emitted varying levels of luminescence, which were measured using a MAGPIX system (Luminex Corporation, Austin, TX, USA). Seven cytokines, encompassing proinflammatory (IL-1β and IL-6) and anti-inflammatory (IL-10 and IL-13) were quantified. Outcomes were reported in picograms per milligrams of tissue (pg/mg).

### 2.9. Serum Corticosterone Determination

Serum corticosterone concentration was assessed using a commercially available ELISA Kit (Abcam, AB108821, Fremont, CA, USA). Optical density was measured at 450 nm using a Microplate Spectrophotometer (Multiskan Go, Thermo Scientific, 51119200, Waltham, MA, USA). Results were expressed as ng/mL. The coefficient of variation was calculated for all samples to assess intra-assay variability. Identical standards and controls were used across all plates to ensure consistency.

### 2.10. Apoptosis Determination

Sagittal sections of the tongue, 20 microns thick, were obtained from the anterior part to the intermolar eminence using a cryostat Leica CM1950 (Wetzlar, Germany). Circumvallate taste buds were collected by cutting the posterior tongue anterior to the foliate papillae at the edge of the oral tongue. Apoptosis determination was performed using the Annexin V-Cy3 apoptosis Kit (Sigma-Aldrich, APOAC-1KT). The determination consists of a double staining with Annexin-Cy3.18 (apoptotic cells in color red) and 6-carboxyfluorescein diacetate (6-CFDA, viable cells in color green). Thus, cells in early apoptosis stages were stained both red and green, appearing orange. The total number of cells (red/orange) was quantified.

### 2.11. Quantification of CB2 Expression

Sagittal sections of the tongue, with a thickness of 20 µm were used. CB2 was labeled by immunohistochemistry. For antigen retrieval, the sections were incubated in a 0.3% sodium citrate solution for 30 min, followed by a round of washes in 0.1 M phosphate-buffered saline (PBS) solution. Endogenous peroxidase activity was blocked by incubating the samples with 10% hydrogen peroxide for 30 min with agitation, followed by three additional washes with 0.1 M PBS. Samples were blocked for 2 with 10% rabbit serum. Section were immunolabeled overnight with a primary antibody anti-CB2, IgG, polyclonal, 1:500, made in rabbit (Thermo Scientific, BS-2377R, Waltham, MA, USA). Then a biotinylated secondary anti-rabbit IgG antibody, 1:2000, made in goat (Thermo Scientific, A16100, Waltham, MA, USA) was incubated by 2 h with a complex of Avidin-Biotin. The final reaction was developed using Diaminobenzidine (DAB), and samples were mounted on glass slides for microscopic analysis. Immunoreactivity was quantified by optical density (OD) using Fiji/ImageJ software version 2.14 (Bethesda, MD, USA), as described by Gómez-Gálvez et al. [33].

### 2.12. Statistical Analysis

Central tendency measures such as median and interquartile range were analyzed. The Kolmogorov–Smirnov Test was performed to assess data normality. Variance was assessed using Levene’s test. Subsequently, ANOVA and Tukey’s post hoc tests were done to compare parametric data, while intergroup differences were assessed using the Kruskal–Wallis test, followed by the Mann–Whitney U test as a post hoc analysis for non-parametric data. The prior level of significance was established at α = 0.05. All the analysis was conducted using GraphPad Software Inc. 9.0.0 (La Jolla, CA, USA). Behavioral Tests were analyzed using ANY-maze video tracking software (Wood Dale, IL, USA). Results were presented as box and whisker plots. Numerical values in the text were expressed as median and interquartile range (IQR). We reported data as median and IQR instead of mean and standard deviation (SD) because potential outliers could have biased measures of central tendency and dispersion. Median and IQR provide a more robust and reliable summary of biological variability.

## 3. Results

### 3.1. β-Caryophyllene Reduces the Deterioration of Health Status

**Figure 2:**
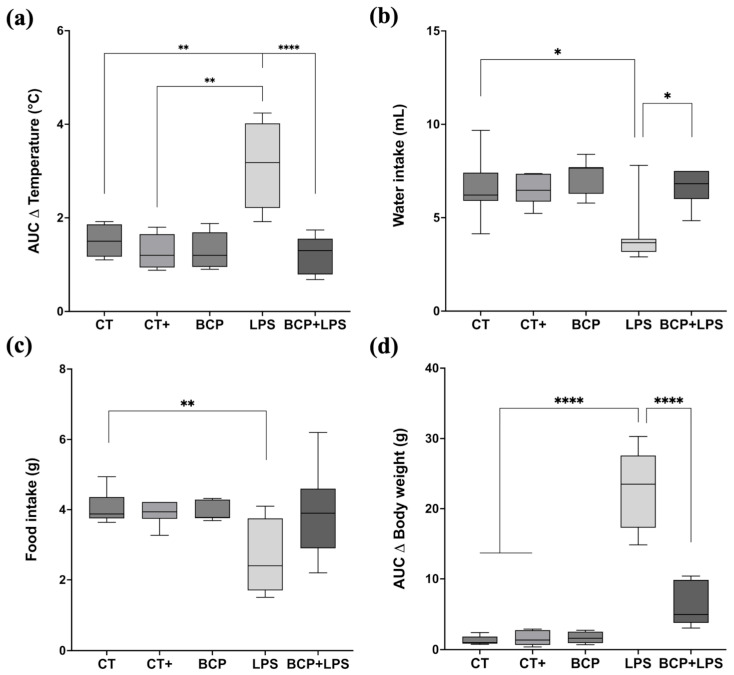
Monitoring of health status (n = 10). Box and whisker plots showing the median values for different experimental groups: Control (CT), Positive Control (CT+), β-Caryophyllene (BCP), Lipopolysaccharide (LPS), and the treatment group (BCP+LPS) (*n* = 10). (**a**) Area under the curve (AUC) of daily average temperature change over seven days. (**b**) Daily average water intake (mL). (**c**) Daily average food intake (g). (**d**) AUC of body weight change (g). Statistical significance is represented as * *p* ≤ 0.05, ** *p* ≤ 0.01, and **** *p* ≤ 0.0001, respectively.

The LPS group (3.18 [1.92–4.24] °C) displayed higher fluctuation in core temperature (Figure 2a), whereas the treatment group (BCP+LPS) did not exhibit drastic temperature shifts (1.37 [0.68–1.74] °C), but rather, it exhibited a core temperature resembling that of the CT group (1.24 [1.10–1.92] °C). Food and water intake (Figure 2b,c) were significantly disrupted in the LPS group. Daily food consumption (2.40 [1.70–3.75] g) was notably lower in comparation to CT group (3.88 [3.75–4.36] g, *p* ≤ 0.05). Water intake also decreased in the LPS group from day two onward and remained diminished through day seven. The BCP+LPS group improved in both food (3.65 [3.40–4.10] g) and water intake (6.82 [6.00–7.50] mL), suggesting that β-Caryophyllene exerted a beneficial effect on the animals’ health status. Regarding body weight (Figure 2d), the LPS group exhibited a median weight difference (Δ) of −4.20 g (−5.10 to −1.70 g). In contrast, the BCP+LPS group showed a less pronounced reduction (−1.50 g), closely resembling the CT group, suggesting a protective effect.

### 3.2. Dysgeusia Improved with β-Caryophyllene Treatment

**Figure 3:**
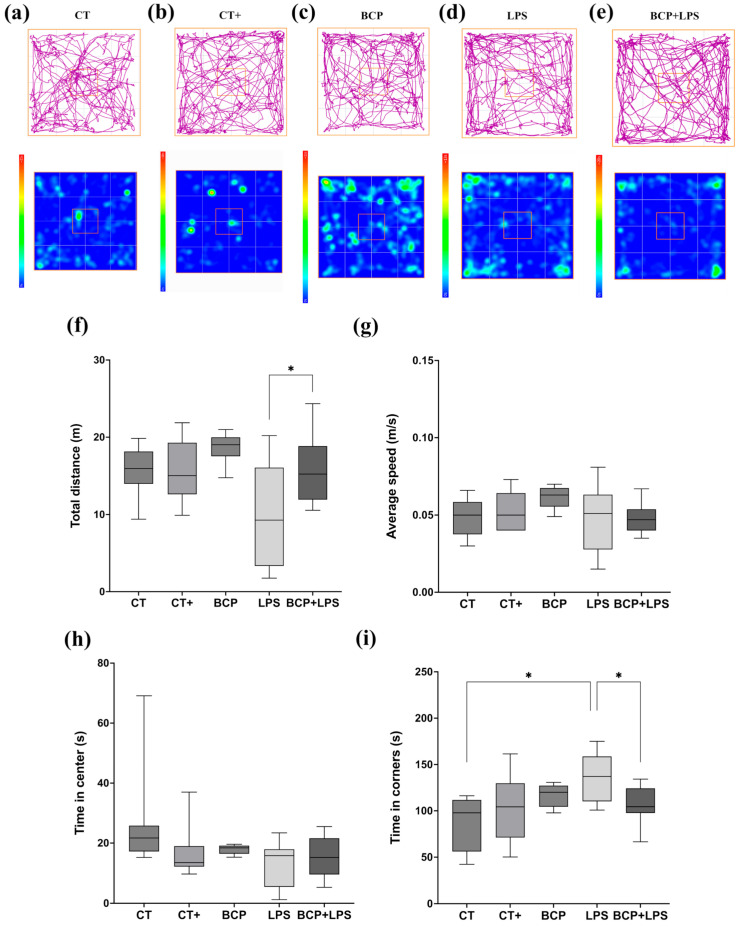
Open field test (n = 10). (**a**–**e**) Representative track maps and heat maps. The orange square on the heat maps marks the center of the open field. (**f**) Box and whisker plot of the total distance traveled by the center of the mouse (m). (**g**) Box and whisker plot of the average speed of the center of the animal (m/s). (**h**) Box and whisker plot of the time spent (s) in the center of the apparatus. (**i**) Box and whisker plot of the time spent in the corner zones (s). Statistical significance is represented as * *p* ≤ 0.05.

In the open field test (Figure 3), mice of the LPS group (Figure 3d) spent more time in the corners (137.00 [110.00–159.00] s) and less time exploring the center of the open field (15.80 [5.45–17.90] s) when compared to the CT group, indicating anxiety-like behavior (Figure 3i) the BCP+LPS group improved the animals’ exploratory behavior, as the time spent in the corners decreased markedly (105.00 [97.70–124.00] s), while the time in the central zone remained similar (15.20 [9.55–21.60] s) (Figure 3h). Locomotor activity (Figure 3f,g) was also affected, with a reduced total distance traveled (9.28 [3.34–16.1] m), in contrast with de BCP+LPS group (15.39 [11.90–18.80] m, *p* ≤ 0.05). Furthermore, the average speed of the animals was not affected by any of the interventions.

**Figure 4:**
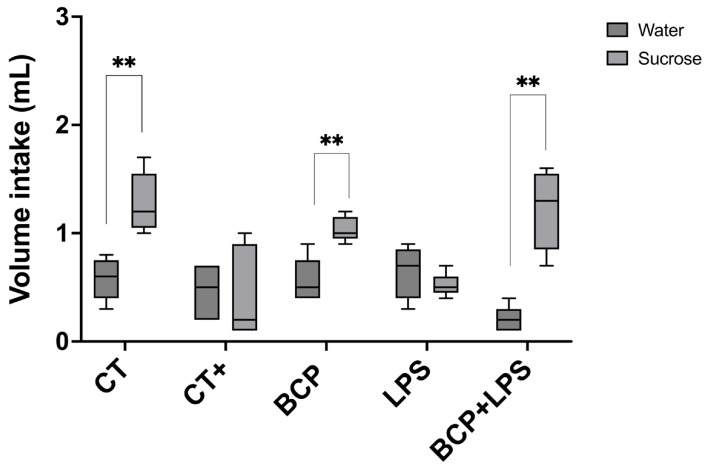
Two-bottle preference test for the evaluation of taste perception (n = 10). Grouped box and whisker plot of water intake (mL, dark gray boxes) and 3% sucrose solution intake (mL, light gray boxes). Statistical significance is represented as ** *p* ≤ 0.01.

To assess the presence of dysgeusia, mice underwent the two-bottle preference test. LPS and the positive control groups showed no significant preference for sucrose solution over water, indicating altered taste perception. Conversely, the treatment group demonstrated a preference for the sweet solution comparable to the control group (*p* ≤ 0.01) (Figure 4). This behavior suggests that β-Caryophyllene improved taste perception.

### 3.3. β-Caryophyllene Attenuates Inflammation and Corticosterone Levels While Modulating the Renin–Angiotensin System

**Figure 5:**
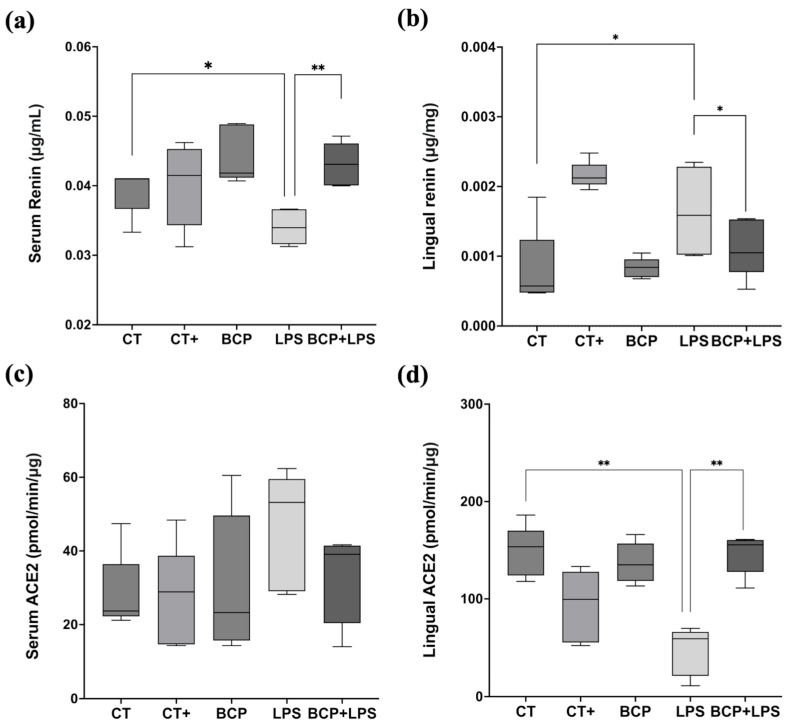
Renin concentration and ACE2 enzymatic activity in serum and tongue (n = 5). (**a**) Box and whisker plot of the serum renin concentration (μg/mL). (**b**) Box and whisker plot of the lingual renin concentration (μg/mg). (**c**) Box and whisker plot of the serum ACE2 enzymatic activity (pmol/min/μg). (**d**) Box and whisker plot of the serum ACE2 enzymatic activity (pmol/min/μg). Statistical significance is represented as * *p* ≤ 0.05 and ** *p* ≤ 0.01.

Serum renin concentration in the LPS group (0.03 [0.02–0.03] μg/mL) exhibited a significant difference with the CT group (0.04 [0.04–0.04] μg/mL, *p* ≤ 0.05) and the BCP+LPS group (0.04 [0.04–0.05] μg/mL, *p* ≤ 0.01) (Figure 5a). On the other hand, renin levels in the lingual parenchyma showed significant differences (*p* ≤ 0.05) between the LPS group (0.0015 [0.001–0.0023] μg/mg) and the treatment group BCP+LPS (0.0011 [0.0015–0.0005] μg/mg, Figure 5b). Regarding serum ACE2 enzymatic activity, no significant differences were found (Figure 5c). However, in lingual parenchyma, it was found that LPS group showed lower activity (59.06 (21.19–66.17) pmol/min/μg) than the BCP+LPS group (155.60 [127.80–160.40] pmol/min/μg, *p* ≤ 0.01) and CT group (153.60 [124.20–170.10] pmol/min/μg, *p* ≤ 0.01) (Figure 5d), indicating that β-Caryophyllene treatment exerted a dual effect, characterized by increased ACE2 activity and reduced renin levels locally in the tongue.

**Figure 6:**
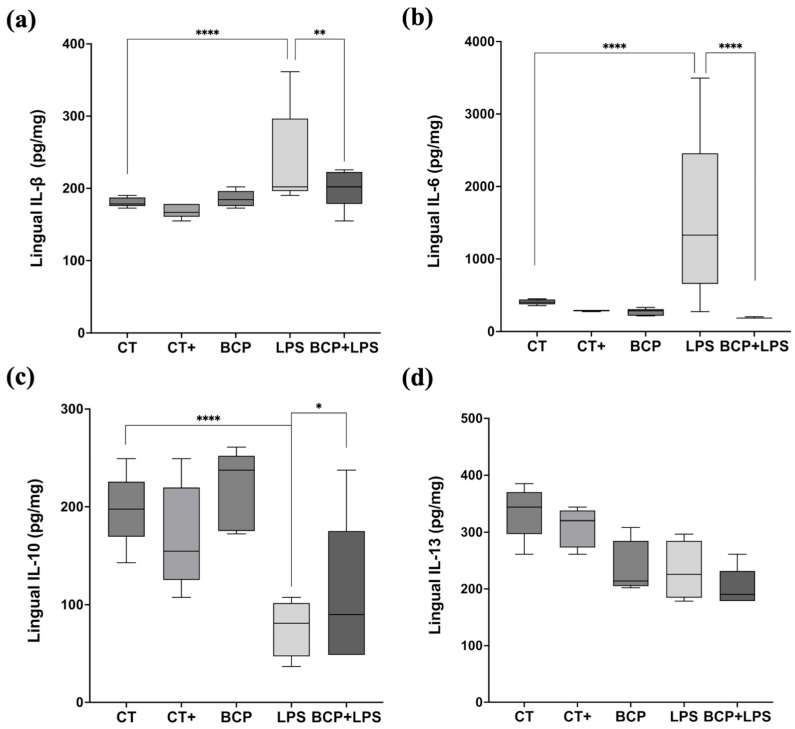
Levels of proinflammatory and anti-inflammatory cytokines in lingual tissue (n = 5). (**a**) Box and whisker plot of the concentration of the proinflammatory cytokine IL-1β (pg/mg). (**b**) Box and whisker plot of the concentration of the proinflammatory cytokine IL-6 (pg/mg). (**c**) Box and whisker plot of the concentration of the anti-inflammatory cytokine IL-10 (pg/mg). (**d**) Box and whisker plot of the concentration of the anti-inflammatory cytokine IL-13 (pg/mg). Statistical significance is represented as * *p* ≤ 0.05, ** *p* ≤ 0.01 and **** *p* ≤ 0.0001.

Cytokine quantification in lingual tissue showed that IL-1β and IL-6 were significantly elevated in the LPS group compared to the CT group (*p* ≤ 0.0001 and *p* ≤ 0.001, respectively). IL-1β levels increased from 178.50 (177.00–185.90) pg/mg in the CT group to 202.10 (199.20–264.10) pg/mg in the LPS group, while IL-6 concentration rose from 190.30 (183.30–197.70) pg/mg in CT group to 237.60 (200.70–295.10) pg/mg in the LPS group. The phenomena indicate an enhanced inflammatory response. Nevertheless, β-Caryophyllene treatment (BCP+LPS) significantly reduced the inflammatory response; IL-1β 184.00 (176.00–196.00) pg/mg, *p* ≤ 0.01, and IL-6 185.00 (184.00–202.00) pg/mg, *p* ≤ 0.0001 (Figure 6a,b).

**Figure 7:**
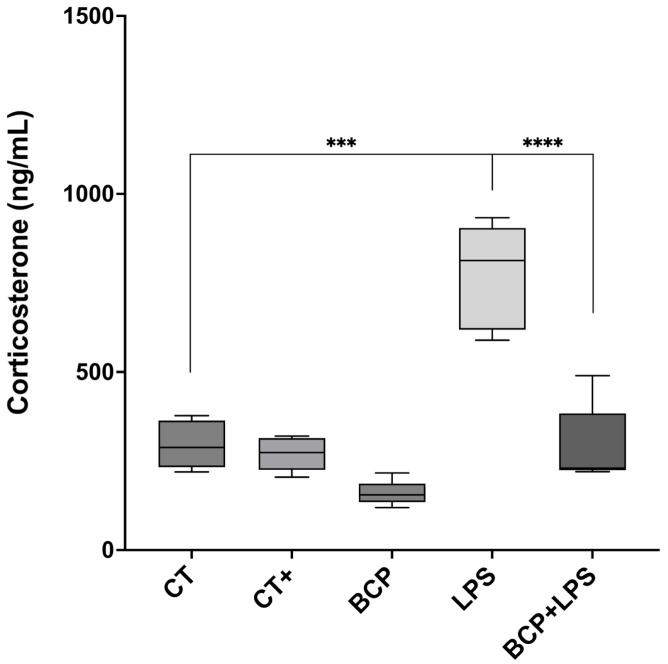
Serum corticosterone concentration (n = 5). Box and whisker plot of the serum concentration of the stress hormone, corticosterone (ng/mL). Statistical significance is represented as *** *p* ≤ 0.001 and **** *p* ≤ 0.0001, respectively.

The anti-inflammatory cytokine IL-10 exhibited a significant increase in the BCP+LPS group (114.00 [94.30–208.00] pg/mg, *p* ≤ 0.05), compared to the LPS group (78.17 [66.73–89.97] pg/mg), indicating that β-Caryophyllene treatment exerted an anti-inflammatory effect (Figure 6c). It is noteworthy that, while IL-13 concentrations did not significantly increase, they showed a positive trend (Figure 6d). Regarding serum corticosterone, there were significant differences between groups (Figure 7). The LPS group exhibited a marked increase (813.00 [619.30–904.00] ng/mL, *p* ≤ 0.001) compared to the CT group (288.00 [233.00–364.00] ng/mL). Notably, treatment with β-Caryophyllene significantly attenuated this response, as corticosterone levels in the BCP+LPS group (230.00 [224.00–384.00] ng/mL) since the serum concentration was lower than in the LPS group (*p* ≤ 0.0001).

### 3.4. β-Caryophyllene Reduces Apoptosis in the Tongue While Preserving CB2 Expression Levels

**Figure 8:**
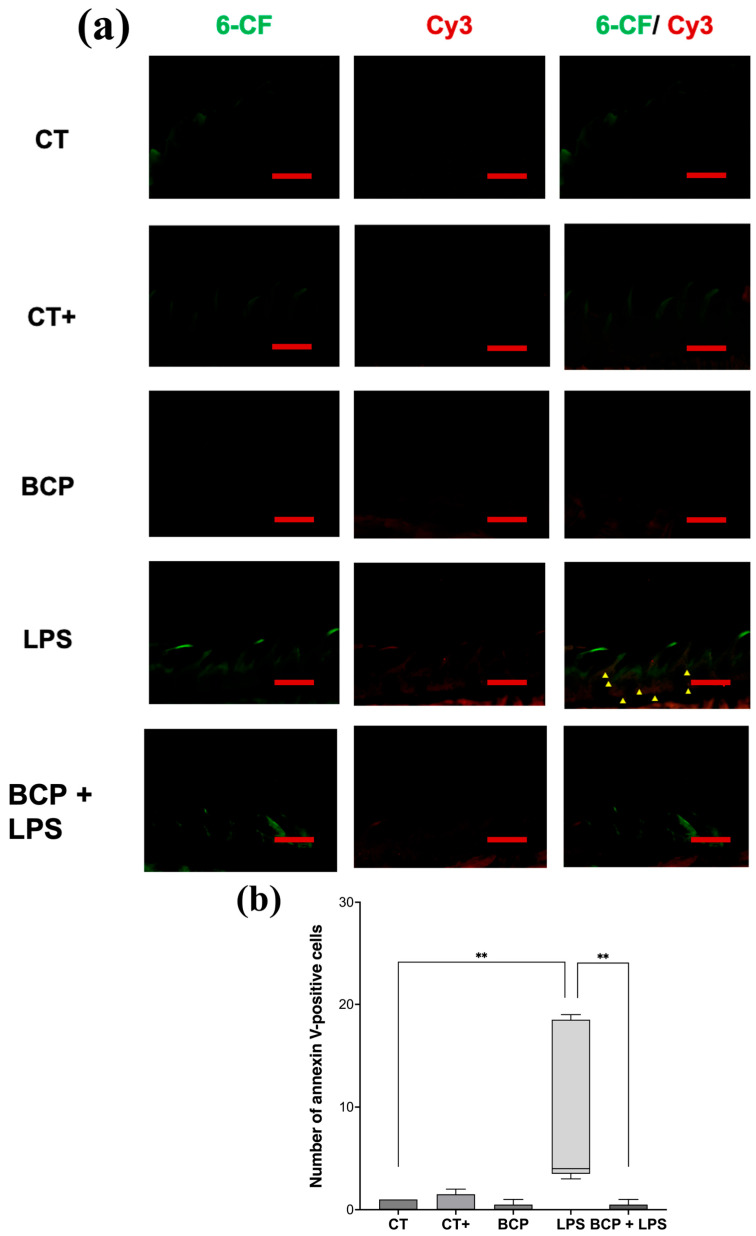
Fluorescence micrographs of annexin V-positive cells in sagittal tongue sections (n = 5). (**a**) Composition of representative micrographs (20×). Viable cells labeled with 6-carboxyfluorescein (6-CF) are shown in green, apoptotic cells labeled with annexin V in red (Cy3), and early-apoptotic cells are shown in orange (6-CF/Cy3). (**b**) Box and whisker plot of apoptotic cell counts (red and orange cells). The scale bar is set at 200 μm. Statistical significance is indicated as ** *p* ≤ 0.01.

Sagittal micrographs of the lingual tissue qualitatively show that both the control groups and the treatment group (BCP+LPS) exhibit a low number of apoptotic cells (red or orange cells), whereas the LPS group display them in abundance (Figure 8a). Quantitative analysis revealed that the LPS group exhibited a markedly elevated number of annexin V-positive cells (4.00 [3.50–18.50], *p* ≤ 0.01) compared to the CT group (0.00 [0.00–1.00]). Notably, treatment with β-Caryophyllene significantly attenuated the number of apoptotic cells relative to the LPS group (0.00 [0.50–1.50], *p* ≤ 0.01) (Figure 8b).

**Figure 9:**
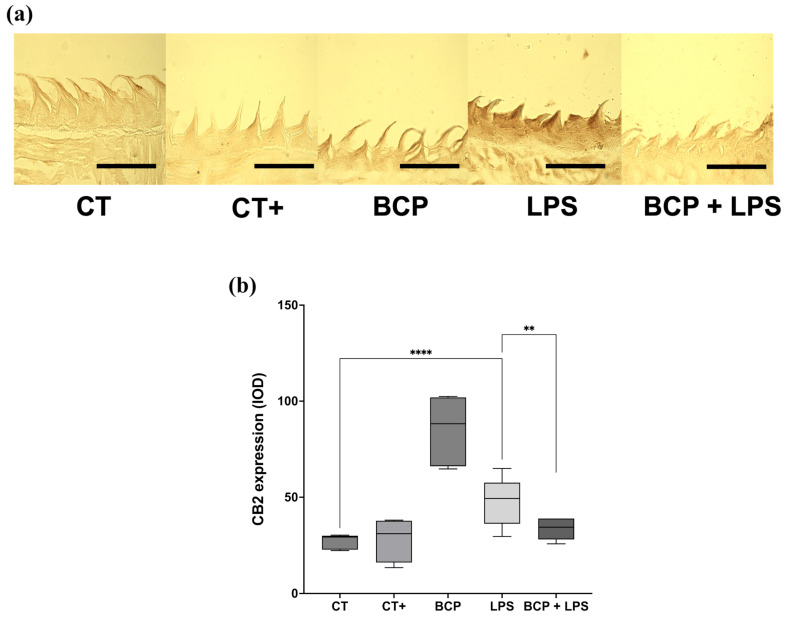
Immunohistochemical labeling of CB2 receptor in sagittal tongue sections (n = 5). (**a**) Composition of representative micrographs (20×). (**b**) Box and whisker plot of the densitometric analysis of CB2 immunoreactivity. The scale bar is set at 200 μm. Statistical significance is represented as ** *p* ≤ 0.01 and **** *p* ≤ 0.0001.

CB2 receptor expression in sagittal tongue sections (Figure 9a) qualitatively revealed greater extension and intensity of immunoreactivity in the LPS group compared with the other groups, including BCP+LPS. Quantitative optical density (OD) analysis confirmed that the LPS group exhibited the highest CB2 expression (49.30 [36.30–57.60], *p* ≤ 0.0001) compared to the CT group (29.20 [22.80–30.00]). Likewise, CB2 expression in the BCP+LPS group was significantly lower than in the LPS group and comparable to that of the control counterparts (34.50 [28.10–39.00], *p* ≤ 0.01) (Figure 9b).

## 4. Discussion

This study evaluated the effect of β-Caryophyllene on the pathophysiology of dysgeusia induced by chronic lipopolysaccharide administration in BALB/c mice by quantifying pro and anti-inflammatory cytokines, analyzing ACE2 enzymatic activity, and assessing both, general health status of the animals and their taste perception. Results support the hypothesis that β-Caryophyllene improves sweet taste perception, reduces inflammation and modulates the local RAS by increasing ACE2 activity and reducing renin concentration. The intraperitoneal administration of lipopolysaccharide in a chronic-persistent manner resulted in inflammatory dysgeusia, as seen in the two-bottle preference test and the cytokine levels. Mice treated with lipopolysaccharide consumed less food and water possibly due to systemic inflammation, malaise and fever associated with endotoxemia [34]. Regarding the decrease in weight loss after lipopolysaccharide insult, a possible explanation lies on the anorexigenic effect of lipopolysaccharide. This phenomenon is characterized by significant lean mass, hunger diminution, and increased catabolic metabolism [35,36].

In contrast, the treatment group (BCP+LPS) exhibited less fluctuation in core temperature and reduced weight loss, suggesting that β-Caryophyllene attenuates the adverse metabolic effects associated with inflammation. Regarding the behavioral trials, the open field test revealed that mice in the LPS group demonstrated reduced exploratory behavior and spent more time in the corners, indicating a potential anxiety-like response [37]. Conversely, the treatment group behaved similarly to the control groups. This phenomenon could be attributed to the improved health status of the animals, which consequently reduced the need to conserve energy by remaining static [38].

It is important to rationalize the range of outcomes regarding the physical condition of the animals following treatment with β-Caryophyllene. Traditionally, β-Caryophyllene has been described as an anti-inflammatory compound and a CB2 receptor agonist [17]. However, it has also been reported that, in the brain, this sesquiterpene could act on glial cells, thereby regulate appetite and the stress response by inducing ghrelin [39] release and reducing corticosterone secretion [20]. The improvement in physiological status could explain the change in exploratory behavior observed in the open field test. In general, better health is synonymous with the absence or mitigation of an underlying inflammatory process, a phenomenon we indeed observed when analyzing proinflammatory cytokine levels. Reduced inflammation and less physical deterioration could lead to a diminished stress response, which is reflected in lower circulating corticosterone levels and a more archetypical behavioral profile. Previous studies, such as that of Bahi et al. (2014) [40] on depression, are consistent with our findings, as in both experimental contexts β-Caryophyllene reduced anxiety-like behaviors and enhanced exploratory activity in the open field test.

The two-bottle preference test is a reliable method for assessing taste alterations [41,42,43], and in the present study, it showed that the administration of β-Caryophyllene did not affect taste perception, while the treatment of the inflammatory insult with β-Caryophyllene improved dysgeusia. Under this observation, β-Caryophyllene may have acted by reducing inflammation in the lingual parenchyma, since it is well established that inflammation alters sensory function including taste perception [44].

Regarding the counter-regulatory axis of the RAS in lingual tissue, the LPS group showed increased renin and decreased ACE2, suggesting deregulation driven by inflammation. Previous studies have demonstrated that renin is expressed in gustatory cells; moreover, Shigemura et al. (2019) [18] found that renin is expressed in the apical regions of the tongue. The authors observed that renin expression was higher in mice subjected to water deprivation, suggesting that renin may be stimulated by microenvironmental factors such as local inflammation and oxidative stress. It is noteworthy to highlight that the renin increase could be deleterious to lingual tissue, as renin positively feeds back the inflammatory response through the (pro)renin receptor (P)RR [45] hence, its reduction after β-Caryophyllene treatment is protective. Moreover, our results showed that treatment with β-Caryophyllene enhanced the enzymatic activity of ACE2. It has been reported that the heptapeptide angiotensin-(1-7) (Ang-(1-7)), a product of ACE2 catalytic activity, exerts anti-inflammatory effects through activation of the Mas receptor [46]. Thus, the increase in ACE2 activity induced by β-Caryophyllene may have contributed to a negative regulation of inflammation. It is also plausible that this downregulation of inflammation led to the observed increase in IL-10 levels within the lingual parenchyma [47]. The main hypothesis regarding how β-Caryophyllene enhances ACE2 enzymatic activity is supported by in silico evidence. It has been reported that a favorable energetic interaction occurs between the sesquiterpene and the hinge domain of the enzyme, resulting in a potentiating effect [27,48].

Our results show that IL-1β and IL-6 levels increased following the lipopolysaccharide. These two cytokines activate the NF-κB and MAPK pathways [49,50], which in turn inhibit the expression of genes associated with gustatory receptors, such as taste receptor type 1 (TAS1R) and taste receptor type 2 (TAS2R), responsible for sweet and bitter taste perception, respectively [51]. The work of Narukawa et al. (2018) [52] demonstrates that aging is associated with the deterioration of taste perception, primarily due to chronic inflammation and the reduction in gustducin, a G protein essential for the proper functioning of TAS1R and TAS2R [53]. Conversely, the observed anti-inflammatory effect and the improvement in ageusia following β-Caryophyllene treatment could be attributed to the inhibition of the NF-κB pathway through activation of its canonical receptor, CB2, as well as to the enhancement of ACE2 enzymatic activity.

Corticosterone concentration increased following the chronic-persistent lipopolysaccharide insult, likely as a stress response mediated by activation of the hypothalamic–pituitary–adrenal (HPA) axis [54]. Proinflammatory cytokines such as IL-1β have been described as endogenous pyrogens acting at the hypothalamic level [55]. In this context, treatment with β-Caryophyllene (BCP+LPS), significantly reduced proinflammatory cytokine levels, likely attenuated HPA axis activation, resulting in lower circulating corticosterone levels. Other studies are consistent with our results. Zoppi et al. (2014) [56] described that the CB2 agonist JWH-133 prevented the stress-induced increase in proinflammatory cytokines such as Tumor Necrosis Factor-α (TNF-α), as well as proinflammatory enzymes like cyclooxygenase-2 (COX-2), thereby mitigating the resulting cellular oxidative and nitrosative damage.

Our results also demonstrate the ability of β-Caryophyllene to reduce apoptosis following inflammation. Supporting evidence has been reported by Alberti et al. (2017) [57], who found that β-Caryophyllene attenuates the inflammatory response by reducing microglial cell activation and modulating the Th1/Treg balance in the context of multiple sclerosis. Moreover, another study further highlights the ability of β-Caryophyllene to suppress apoptosis induced by oxidative stress [58]. Furthermore, histological analysis of CB2 expression revealed that CB2 levels in tongue sections were markedly higher in the LPS group compared to both the control and treatment groups. These findings are consistent with previous reports indicating that, following an inflammatory insult mediated by Toll-like receptors (TLR), CB2 expression increases dramatically, possibly as part of a negative feedback mechanism aimed at controlling inflammation [59]. Therefore, the absence of this CB2 upregulation in the BCP+LPS group suggests that the inflammatory condition in the lingual tissue was not sufficiently severe to trigger receptor overexpression.

The main limitations of this study include the absence of CB2 antagonists, such as AM-630, to determine whether the modulation of the RAS is CB2-dependent. Additionally, the use of wild-type and ACE2 knockout (ACE2KO) mice would allow for a deeper understanding of the direct interaction between ACE2 and β-Caryophyllene. Another limitation of the study lies in the two-bottle preference test, as this behavioral paradigm employs sweet solutions, given that they constitute a rewarding stimulus. In contrast, the use of sour, bitter, or salty flavors induces aversion and avoidance; therefore, the loss of perception of these other tastes cannot be determined. Future studies should explore the molecular mechanisms underlying these effects, particularly the interaction between CB2 receptor activation and ACE2 potentiation, to better elucidate the therapeutic potential of β-Caryophyllene in taste disorders and other inflammation-driven conditions.

## 5. Conclusions

This study demonstrates that β-Caryophyllene improves dysgeusia by exerting significant anti-inflammatory and cytoprotective effects in a murine model. Chronic lipopolysaccharide administration disrupted taste perception, elevated corticosterone levels, increased apoptosis in lingual tissue, and upregulated CB2 receptor expression, reflecting a deep inflammatory state. In contrast, treatment with β-Caryophyllene mitigated these alterations by reducing proinflammatory cytokines, normalizing corticosterone levels, decreasing taste cell apoptosis, and preventing CB2 overexpression. Furthermore, the observed increase in ACE2 enzymatic activity, along with the reduction in renin levels, suggests that β-Caryophyllene modulates the RAS, thereby promoting an anti-inflammatory environment in the lingual tissue. These findings highlight the potential of β-Caryophyllene as a modulator of RAS and a protective agent against taste dysfunction associated with chronic systemic inflammation like dysgeusia.

## Data Availability

Datasets for this study are available from the corresponding author upon request.

## Abbreviations

The following abbreviations are used in this manuscript:

6-CFDA	6-carboxyfluorescein diacetate
(P)RR	(Pro)Renin Receptor
Ang-(1-7)	Angiotensin-(1-7)
Ang I	Angiotensin I
Ang II	Angiotensin I
AT1R	Angiotensin II type 1 Receptor
AT2R	Angiotensin II type 2 Receptor
ACE	Angiotensin-Converting Enzyme
ACE2	Angiotensin-Converting Enzyme 2
ACE2KO	Angiotensin-Converting Enzyme 2 Knockout
AUC	Area Under the Curve
BCP	β-Caryophyllene control group
BCP+LPS	Treatment group
CB2	Cannabinoid Receptor type 2
CIBO	Centro de Investigación Biomédica de Occidente
COX-2	Cyclooxygenase-2
CT	Control group
CT+	Positive control group
Cy3	Cyanine 3
DAB	Diaminobenzidine
HPA	Hypothalamic–Pituitary–Adrenal axis
IL-1β	Interleukin-1β
IL-6	Interleukin-6
IL-10	Interleukin-10
IL-13	Interleukin-13
IMSS	Instituto Mexicano del Seguro Social
IQR	Interquartile Range
LPS	Lipopolysaccharide group
MasR	Mas Receptor 1
MAPK	Mitogen-Activated Protein Kinases
NCD	Noncommunicable Chronic Diseases
NF-κB	Nuclear Factor-kappa B
OD	Optical Density
RAS	Renin–Angiotensin System
SD	Standard Deviation
TLR	Toll-Like Receptors
TNF-α	Tumor Necrosis Factor-α
ZnSO4	Zinc Sulphate
